# Genomic analysis of the molecular neuropathology of tuberous sclerosis using a human stem cell model

**DOI:** 10.1186/s13073-016-0347-3

**Published:** 2016-09-21

**Authors:** Nils Grabole, Jitao David Zhang, Stefan Aigner, Nadine Ruderisch, Veronica Costa, Felix C. Weber, Michel Theron, Nikolaos Berntenis, Olivia Spleiss, Martin Ebeling, Gene W. Yeo, Ravi Jagasia, Anna Kiialainen

**Affiliations:** 1 Roche Pharma Research and Early Development, Pharmaceutical Sciences, Roche Innovation Center Basel, F. Hoffmann-La Roche Ltd, Grenzacherstrasse 124, Basel, 4070 Switzerland; 2 Department of Cellular and Molecular Medicine, Institute for Genomic Medicine, UCSD Stem Cell Program, Sanford Consortium for Regenerative Medicine, University of California at San Diego, La Jolla, CA 92037 USA; 3 Roche Pharma Research and Early Development, Neuroscience, Ophthalmology and Rare Diseases, Roche Innovation Center Basel, F. Hoffmann-La Roche Ltd, Grenzacherstrasse 124, Basel, 4070 Switzerland

**Keywords:** Astrocytes, mTOR, Stem cell disease modeling, TSC2, Tuberous sclerosis

## Abstract

**Background:**

Tuberous sclerosis complex (TSC) is a genetic disease characterized by benign tumor growths in multiple organs and neurological symptoms induced by mTOR hyperfunction. Because the molecular pathology is highly complex and the etiology poorly understood, we employed a defined human neuronal model with a single mTOR activating mutation to dissect the disease-relevant molecular responses driving the neuropathology and suggest new targets for treatment.

**Methods:**

We investigate the disease phenotype of TSC by neural differentiation of a human stem cell model that had been deleted for *TSC2* by genome editing. Comprehensive genomic analysis was performed by RNA sequencing and ribosome profiling to obtain a detailed genome-wide description of alterations on both the transcriptional and translational level. The molecular effect of mTOR inhibitors used in the clinic was monitored and comparison to published data from patient biopsies and mouse models highlights key pathogenic processes.

**Results:**

*TSC2*-deficient neural stem cells showed severely reduced neuronal maturation and characteristics of astrogliosis instead. Transcriptome analysis indicated an active inflammatory response and increased metabolic activity, whereas at the level of translation ribosomal transcripts showed a 5’UTR motif-mediated increase in ribosome occupancy. Further, we observed enhanced protein synthesis rates of angiogenic growth factors. Treatment with mTOR inhibitors corrected translational alterations but transcriptional dysfunction persisted.

**Conclusions:**

Our results extend the understanding of the molecular pathophysiology of TSC brain lesions, and suggest phenotype-tailored pharmacological treatment strategies.

**Electronic supplementary material:**

The online version of this article (doi:10.1186/s13073-016-0347-3) contains supplementary material, which is available to authorized users.

## Background

The mTOR pathway is involved in a plethora of pathologies, in particular carcinogenesis [1]. However, the large number of processes downstream of mTOR and resulting heterogeneity of clinical manifestations impede a deeper understanding of the disease mechanisms and selection of the optimal treatment strategy. For example, patients with tuberous sclerosis complex (TSC), caused by mutations in the tumor suppressor genes *TSC1* or *TSC2* leading to mTOR hyperfunction, show heterogeneity of benign tumors and cellular dysplasia in multiple organs, including astrocytomas and cortical tubers in the brain [2–4]. Loss of heterozygosity for either *TSC* gene due to somatic mutation of the functional allele in heterozygous patients was detected in these lesions and induces cancerous growth [5–7]. In addition, TSC patients develop central nervous system abnormalities, including structural alterations of the cortex, epilepsy, and psychiatric symptoms [8]. Clinical trials with mTOR inhibitors are ongoing to treat the manifestations of this disease [9, 10]. However, while mTOR inhibitors have tremendous potential as disease modifying agents, it remains unclear if they can be effective to treat the full spectrum of TSC-associated pathophysiology.

Work on mouse models identified neural progenitor cells as the origin of brain lesions [11–15]. Nonetheless, the paucity of human cellular models has limited a better mechanistic understanding of brain lesions in TSC patients. Hence, availability of a human TSC in vitro system to model the in vivo pathogenesis and perform experimental analysis would enable discovery of novel targets for pharmacological intervention. Recently a pioneering study on osteosarcoma demonstrated the utility of modeling carcinogenesis with human stem cells to elucidate disease mechanisms and identify new treatment options [16].

Here we used human neural stem cells (NSCs) derived from embryonic stem cells (ESCs) that have been biallelically deleted for *TSC2* by genome editing to study the cellular and molecular pathophysiology of TSC. This TSC in vitro model showed reduced neuronal maturation potential and increased commitment to the astrocyte lineage, providing valuable insight for the study of TSC patient biopsies [17]. Using RNA sequencing (RNA-Seq) and ribosome profiling, we performed a comprehensive analysis of the genome-wide consequences of *TSC2* loss on both transcription and translation. We detected a disease-relevant inflammatory response on the transcriptional level while translatome analysis demonstrated motif-dependent translational dysfunction of protein synthesis factors as well as increased production of angiogenic growth factors. Inhibition of mTOR signaling corrected the translation defects but not the inflammatory or angiogenic growth factor response, which were due to altered transcription. Thus we provide important insight into the molecular pathology of tuberous sclerosis and present an experimental system for future investigation of disease-modifying compounds beyond mTOR inhibitors and development of comprehensive therapies for TSC.

## Methods

### Cell line generation and neural differentiation

An allelic deletion series of *TSC2* was established from the parental ESC line SA001 (NIH registration number 0085) by use of zinc finger nucleases targeting exon 11 of the *TSC2* locus. Site-specific integration was confirmed by polymerase chain reaction (PCR) amplification of the genomic locus followed by direct sequencing. Absence of non-specific integration sites was determined by targeted locus amplification followed by deep sequencing. Neural conversion of ESCs to NSCs was performed using a dual SMAD inhibition protocol. Generation of cell lines is described and documented in detail by Costa et al. [18].

NSCs were cultured according to standard methods. All used tissue culture dishes were coated with poly-L-ornithine (Sigma Aldrich) and laminin (Roche) and undifferentiated cultures were maintained in a basic medium composed of a 1:1 mix of DMEM:F12 Glutamax medium and Neurobasal medium (both Gibco, Invitrogen) that was supplemented with 1× B27, 1× N2, and 0.1 mM beta-mercaptoethanol (all Gibco, Invitrogen). For self-renewing conditions the following growth factors were added: 10 ng/mL FGF2, 20 ng/mL BDNF (both Peprotech), and 10 ng/mL EGF (R&D Systems). Ventralization was induced for a period of seven days by replating the cells at a density of 12,000 cells/cm^2^ and changing the supplementing growth factors to 200 ng/mL Shh, 100 ng/mL FGF8 (both Peprotech), and 100 μM ascorbic acid phosphate (Sigma Aldrich). Neuronal differentiation was initiated by replating the cells at a density of 40,000 cells/cm^2^ in basic medium supplemented with 20 ng/mL BDNF, 10 ng/mL GDNF (both Peprotech), 0.5 mM cAMP (BIOLOG Life Science), and 100 μM ascorbic acid phosphate (Sigma Aldrich). Medium was changed twice per week until the day of analysis.

### Library preparation and sequencing

Ribosome profiling and RNA sequencing libraries were prepared using the TruSeq Ribo Profile kit (Illumina, #RPHMR12126) as detailed in the manufacturer’s protocol. Cells for each biological replicate of control, heterozygous, and homozygous cells with or without drug treatment were washed with ice cold PBS and lysed on ice in the presence of 100 μg/mL cycloheximide (Sigma). The cleared lysate was flash frozen and stored at −80 °C until further processing. For ribosomal RNA depletion, the RiboZero magnetic Gold kit (Illumina) was used. Quality of amplified libraries was accessed by capillary electrophoresis with a high sensitivity DNA chip on a 2100 Bioanalyzer (Agilent Technologies) and quantified by quantitative PCR with a sequencing library quantification kit (KAPA Biosystems) on a Roche Light Cycler 480. Multiplexed libraries with 1 % spiked in PhiX control were sequenced on a HiSeq2500 instrument for 50 cycles using version 4 chemistry reagents (Illumina). BCL files were converted to the fastq format for further bioinformatics processing.

The sequencing data from this publication is available at the GEO database.

### Bioinformatic analysis

Linker tags were removed from RNA-Seq and ribosome profiling reads by the FASTX Toolkit, v0.0.13 (http://hannonlab.cshl.edu/fastx_toolkit/). All reads that mapped to rRNAs, tRNAs, or mitochondrial rRNAs were removed, and the remaining reads were mapped to RefSeq (v38) by TopHat v2.0.13 [19]. Finally, all read counts that mapped uniquely to genes were extracted for expression analysis with the help of samtools, v1.1 [20].

We applied the *edgeR* [21] algorithm for differential gene expression analysis and identified genes with absolute log2 fold change larger than 1 and Benjamini–Hochberg adjusted *p* value smaller than 0.05 as significantly changed. We applied the *camera* [22] algorithm for gene set enrichment analysis with gene sets collected in the Roche internal database RONET. The same method was applied for upstream regulator analysis, with transcriptional targets of human transcription factors and gene expression modulators curated from literature in RONET as input. Gene ontology enrichment analysis was performed using the Fisher’s exact test.

To validate our findings with results of previous studies, we retrieved gene expression data from GEO (GSE35338 for Zamanian et al. and GSE16969 for Boer et al.) or ArrayExpress (E-MEXP-2351 for Tyburczy et al.) and performed differential expression analysis with the *limma* [23] package. Gene set enrichment analysis was performed in the same way as RNA-Seq data to allow fair comparisons.

We constructed an analysis pipeline to analyze the ribosome profiling data. In essence, the pipeline estimates both amplitude and statistical significance of differential translation efficiency (TE). TE is defined as the ratio between expression levels measured by messenger RNA sequencing and by ribosome sequencing, with pseudo-counts of 1 added to each to avoid division by zero. The amplitude of TE change between two conditions is estimated by the log2-transformed ratio of two TEs. The statistical significance is estimated by the *babel* method [24] based on errors-in-variables regression models.

### Microfluidic quantitative PCR (qPCR)

Cells from biological triplicates were harvested in TRIzol Reagent (LifeTechnologies) in weekly intervals and RNA was extracted according to the manufacturer’s instructions. Traces of contaminating DNA were removed using the TURBO DNA-free kit (LifeTechnologies). Synthesis of complementary DNA (cDNA) was performed using the SuperScript III First Strand Synthesis Mix (LifeTechnologies). For the non-enzyme control, a reference pool of all samples was used. cDNAs were subjected to specific target amplification prior to qPCR analysis using a pool of standard predesigned TAQman assays to be used later (Applied Biosystems) with a pre-amplification mastermix (Roche Life Science). Preamplified cDNA was diluted 1:5 and processed together with the TAQman assays for analysis in 96.96 Dynamic Array integrated fluidic chips on the BioMark HD platform (Fluidigm) according to the manufacturer’s instruction. Data were analyzed using Real-Time PCR Analysis software (Fluidigm).

### Flow cytometry

For flow cytometry analysis, cells were analyzed as described previously [25]. In brief, cells were dissociated with Accutase (Sigma, A6964), treated with DNase (Sigma, D5025), and resuspended in sorting medium (growth medium supplemented with 0.5 % BSA and 5 mM EDTA). 1 × 10^6^ cells were stained with anti CD184 (BD555976) and anti CD44 (BD555479) antibodies according to the manufacturer’s recommendations for 20 min at 4 °C. Stained cells were washed once more with sorting medium and centrifuged at 250 g for 5 min before acquisition of data on a FACSCanto II flow cytometer. For analysis of protein synthesis rates, the Click-iT Plus OPP kit (LifeTechnologies, C10456) was used and cells prepared according to the manufacturer’s instructions. Data were analyzed using FlowJo v10.0.8 software.

### Western blot analysis and protein arrays

Cells for protein analysis were directly lysed using RIPA buffer (LifeTechnologies) supplemented with protease and phosphatase inhibitors (Roche Life Science). Total protein concentration was quantified by BCA assay (LifeTechnologies). For SDS-PAGE, 20 μg of total protein lysate were analyzed by western blotting on nitrocellulose membranes (BIO-RAD). For western blot analysis, all antibodies were used at 1:1000 (except anti-*β*-actin which was used 1:20,000) and chemiluminescence was detected with ECL Reagent (GE Healthcare) using a Fusion FX system (Vilber Lourmat). The same membranes used to detect proteins of interest were stripped, blocked, and reprobed with anti-beta-Actin antibody. Primary antibodies used were mouse-anti-STAT3 (CST #9139), rabbit-anti-phospho-STAT3 (Tyr705) (CST #9131), rabbit-anti-GFAP (Dako Z0334), HRP linked mouse-anti-beta-Actin (Abcam ab49900), rabbit-anti-S6 ribosomal protein (CST #2217), and rabbit-anti-phospho S6 ribosomal protein (Ser240/244) (CST #2215). Secondary antibodies used were HRP linked anti-mouse (Santa Cruz Biotechnology sc-2005) and HRP linked anti-rabbit (Santa Cruz Biotechnology sc-2030). For quantification of cytokines and angiogenesis factors, the secreted proteins in conditioned media were detected using Proteome Profiler array kits (R&D Systems) according to the manufacturer’s instructions. The intensity of the signals was quantified and normalized by densitometry using ImageJ software.

### Immunocytochemistry

For immunofluorescence analysis, cells were cultured in 96-well plates (BD Falcon), fixed in 4 % PFA in PBS for 10 min, permeabilized with 0.25 % (v/v) Triton X-100 in PBS for 5 min, and incubated for 45 min at room temperature in blocking solution (10 % BSA w/v in PBS). Primary (anti-GFAP DAKO, anti-HuC/D Life Technologies, anti-Doublecortin Santa Cruz) and secondary antibodies were diluted in 3 % BSA (w/v) and incubated overnight at 4 °C and for 1.5 h at room temperature, respectively. Upon removal of secondary antibody, cells were incubated with DAPI (1 μg/mL) in PBS for 5 min at room temperature. Confocal fluorescence images were acquired using a Leica TCS SP5 (Leica Microsystems) inverted microscope. Stacks acquired along the z-axis were converted into maximum projections using LAS-AF software.

## Results

### *TSC2*-deficient neural stem cells exhibit altered neuronal and glial differentiation

Loss of heterozygosity was reported in TSC patient biopsies and mouse models showed that brain lesions arise from neural progenitor cells [6, 12]. In order to investigate the pathogenesis of these brain lesions, we employed forebrain neural stem cells derived from human embryonic stem cells as an in vitro model. Two *TSC2*-deficient cell lines were derived from a parental stem cell line by performing genome editing using zinc finger nucleases to disrupt both *TSC2* alleles as described by Costa et al*.* [18]. In control cell lines, the same inactivating cassette was inserted into the *AAVS1* locus, which induces no phenotypic effects [26], and absence of off-target integrations was determined by targeted locus amplification sequencing. In contrast to the control cell lines, *TSC2*-deficient cells showed no detectable TSC2 protein expression and increased phosphorylation of mTOR target ribosomal protein S6 (RPS6), indicating mTOR hyperfunction (Additional file 1: Figure S1A).

**Fig. 1:**
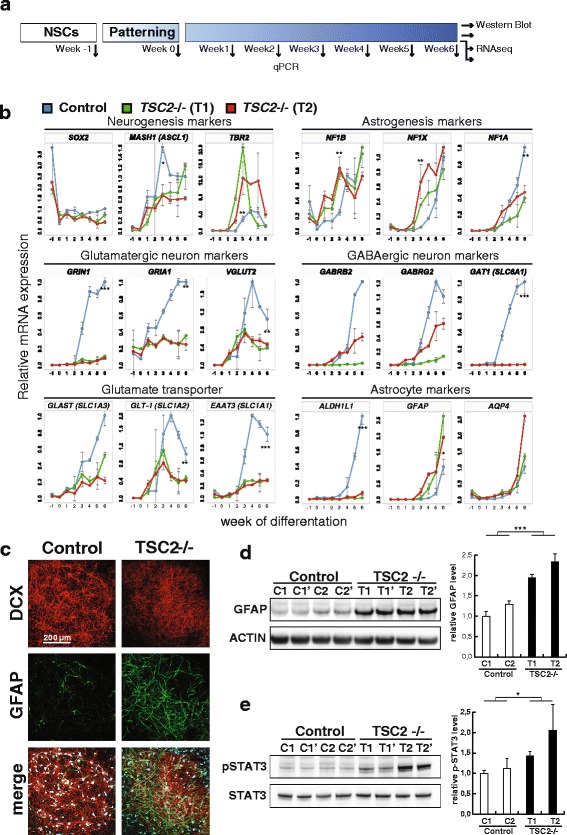
Reduced functional maturation in the absence of *TSC2*. **a** *Experimental protocol outline*. For an experiment neural stem cells, generally maintained in self-renewing conditions (week −1), were subjected to a patterning step for seven days (week 0) before induction of neural differentiation (week 1–6). Samples were collected in weekly intervals for gene expression quantification by qPCR. After six weeks of differentiation, additional samples were collected for western blot, immunofluorescence, RNA-Seq, and ribosome profiling. **b** Relative quantification of expression levels of the indicated neural marker genes over the course of six weeks of differentiation in control and *TSC2* deleted cell lines. Expression levels in control cells after six weeks of differentiation are normalized to 1. *Error bars* show standard error of the mean and *asterisks* indicate significant difference in a two-tailed Student’s t-test, *p < 0.05, **p < 0.01, ***p < 0.001. **c** Representative confocal *images* of 6-week-old neural cultures of control and *TSC2* deleted cell lines stained for doublecortin (DCX) and GFAP. Confocal *z-axis* stacks were acquired and restructured. **d**, **e** *Western blot analysis* for the indicated proteins of neural cultures after six weeks of differentiation using independent derived control (C1, C2) and *TSC2* deletion (T1, T2) cell lines in biological replicates (e.g. C1 and C1’). Densitometric analysis of GFAP protein and STAT3 phosphorylation levels is normalized to beta Actin and total STAT3 levels, respectively. Data are normalized to the level in control cell line C1 and *error bars* represent standard deviation. Significance is determined by Student’s two-tailed t-test, *p < 0.05, ***p < 0.001

We applied a differentiation protocol that induces a temporal order of neurogenesis and gliogenesis similar to in vivo neurodevelopment with cells acquiring expression of markers of functional neural cell types as well as mature electrophysiological properties within six weeks of differentiation. Reproducibility of the protocol was shown by the high degree of correlation of protein expression changes after 41 days of differentiation across different wild-type NSC lines with at least five independently differentiated biological replicates [27]. To monitor cell fate over the course of neuronal differentiation, we collected samples in weekly intervals (Fig. 1a) and determined transcript levels of neuronal and glial differentiation and maturation markers by qPCR (Fig. 1b and Additional file 1: Figure S1B). At the start of differentiation, *TSC2*-deficient cells downregulated neural stem cell marker *SOX2* similar to control cells. However, at week 3 of differentiation, pro-neural transcription factor *MASH1* was expressed at significantly lower levels in *TSC2*-deficient cells. This suggests that generation of functional neurons might be affected, although at the same time induction of *TBR2*, associated with a neuronal progenitor state, was observed. In contrast to neuronal fate regulator *MASH1*, the glial cell fate regulators *NF1B* and *NF1X* were expressed earlier and stronger as well.

Eventually, at week 6, *TSC2*-deficient cultures exhibited in comparison to controls severely reduced levels of transcripts required for functional maturation of glutamatergic and GABAergic neurons. These include both pre- and postsynaptic markers, such as GABA and glutamate receptors, and neural cell adhesion proteins. Equally, markers of oligodendrocytes (*OLIG1*, *OLIG2*) and astrocytes (*ALDH1L1*, *S100B*) as well as other critical genes for astrocyte functionality like glutamate transporters (*EAAT3*, *GLAST*, and *GLT-1*) were strongly downregulated, together indicating that *TSC2* is required for generation of functionally mature cell types.

A reduced differentiation of radial glia cells into oligodendrocytes leading to myelination defects is also seen in the TSC mouse models [28–30]. Simultaneously the majority, but not all, of these models show a strong upregulation of GFAP as a marker of immature glia and astrogliosis [28, 29, 31], which can also be found in TSC patient biopsies [32]. Notably, such markers (*GFAP*, *AQP4*) also showed stronger expression in our *TSC2*-deficient cells (Fig. 1b), suggesting an altered cell fate also in the in vitro model. This was also supported by flow cytometry analysis of CD44 and CD184 (Additional file 1: Figure S1C), which can monitor the generation of neuronal and glial populations from human pluripotent cells [25]. Therefore, we confirmed GFAP expression at the protein level by immunofluorescence staining, revealing a stark increase in the number of cells expressing GFAP (Fig. 1c and Additional file 1: Figure S1D). Furthermore, western blot analysis showed that there was a significant increase in GFAP protein levels in *TSC2*-deficient cultures (Fig. 1d). As STAT3 signaling is linked to GFAP expression and involved in regulation of astrocyte development and phenotype [33, 34], we determined STAT3 activity by western blot analysis and detected a significant increase in phospho-STAT3 levels in our *TSC2*-deficient cell lines (Fig. 1e).

In summary, using our human cellular model, we showed that differentiating *TSC2*-deficient NSCs exhibit increased expression of markers indicative of an early astroglial lineage and reduced differentiation in mature neuronal and astroglial cell types.

### Transcriptome analysis illustrates an active inflammatory response in the *TSC2* brain lesion model

**Fig. 2:**
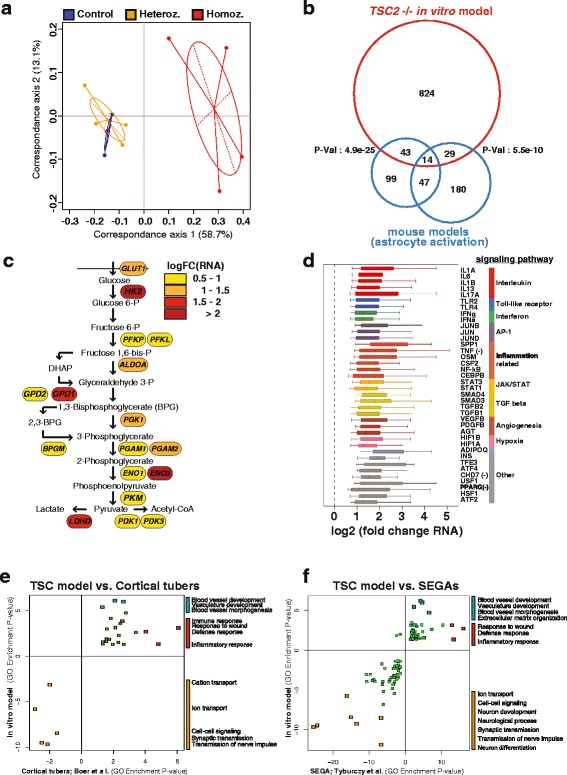
Transcriptional signatures of inflammation and angiogenesis mark the molecular pathology of TSC. **a** *Correspondence analysis* of RNA-Seq results from control (*blue*), *TSC2* heterozygous (*orange*), and homozygous mutant (*red*) cell lines after six weeks of differentiation. Each *dot* represents a biological replicate. **b** *Venn diagram* showing overlap of genes deregulated in two mouse models of astrocyte activation (*blue*) [36] with those in *TSC2*-deficient cells (*red*). Significance of overlap between gene lists (*p* values of 4.9E-25 and 5.5E-10) was determined by Fisher’s exact test. **c** *Pathway map* for glycolysis enzymes indicating log2 fold changes of transcript levels in *TSC2*-deficient cells. **d** *Whisker box plot* for target gene expression changes of listed upstream regulators/signaling pathways that were found significantly (false discovery rate (FDR) < 1E-4) and consistently over-expressed in *TSC2*-deficient cells. *Vertical lines* (median); *error bars* (1.5 times the interquartile range); (−): negatively regulated targets. **e**, **f** Gene Ontology gene set enrichment in in viv*o* (*x-axis*) and in vitro (*y-axis*) samples. Gene set analysis based on Fisher’s exact test was applied to the comparison of cortical tubers versus normal cortex autopsies from controls (Boer et al. [37]) or SEGAs versus periventricular control tissue from non-TSC patients against *TSC2*-deficient cells versus control cells. Genes that are significantly differentially expressed (cutoff: FDR < 0.05) are tested for over-representation of Gene Ontology biological process terms. The *p* values are log10 transformed and given a sign according to the direction of gene expression changes in the absence of *TSC2*. Only significantly enriched terms (*p* < 0.05) are reported and no extra filtering was applied. All changes that we observed were consistent in both conditions. Selected points are text labeled; numerical values and labels of all points can be found in the accompanying source data

To characterize the aberrant cell state due to absence of *TSC2*, we performed RNA-Seq on the cells after six weeks of differentiation. We included two *TSC2* heterozygous cell lines derived from the same parental line in the analysis to serve as cellular models recapitulating heterozygous tissue of TSC patients and to study *TSC2* gene dosage dependent effects. While only ten genes showed significant expression changes in *TSC2* heterozygous cells, there were over 2000 genes either upregulated or downregulated in *TSC2* depleted cells (Additional file 2: Figure S2A and Additional file 3: Table S1). Correspondence analysis of transcriptomes, which is conceptually similar to principal component analysis, yet more suitable for discrete sequencing counts [35], illustrated the similarity between control and heterozygous cells as well as the dissimilarity of those to *TSC2* homozygous cells (Fig. 2a).

We aimed to further explore the astrocyte phenotype in *TSC2*-deficient cultures that was suggested by the altered expression of astrocytic markers. Therefore, we compared our RNA-Seq data to published gene expression data of mouse models of astrogliosis, which show strong activation of astrocytes [36]. There was a highly significant overlap of misregulated genes among the datasets as determined by Fisher’s exact test (*p* = 4.9e-25 and 5.5e-10, respectively), despite the overall lower number of genes identified by microarray (Fig. 2b). Gene set enrichment analysis (Additional file 2: Figure S2B–F) identified many induced genes to be implicated in metabolic pathways. In particular the enzymes of almost all catalytic steps in the glycolytic pathway were induced, highlighting an increased metabolic activity in the absence of *TSC2* (Fig. 2c). Moreover, the enrichment in inflammation-related functional categories (Additional file 4: Table S2) supports the notion of an altered astrocyte state.

To identify activation of which signaling pathways could induce the transcriptional changes observed, we performed upstream regulator analysis with our complete dataset. In particular, inflammatory cytokine signaling was highlighted, for example by interleukins and interferons (Fig. 2d); together with transcription factors, such as AP1 and NFkB, which are key mediators of the transcriptional response to inflammation. Also STAT3 activation was identified here again, consistent with the higher STAT3 phosphorylation levels detected in western blot analysis. Other interesting pathways with significant enrichment were related to hypoxia and angiogenic growth factors like VEGF and PDGF.

We addressed the clinical relevance of these findings by comparison with published microarray data from resected cortical tubers of TSC patients [37]. Notably, there was a strong correlation in gene ontology term enrichments for our in vitro model with those for the biopsies (Fig. 2e and Additional file 5: Table S3). Inflammation-related terms showed enrichment in both datasets, such as immune response with a *p* value of 0.0028 for patient tubers and 0.00014 for the in vitro model, thereby attesting relevance to our findings. Shared upregulated genes included interferon-inducible genes, such as *GBP1* and *GBP2*. Also, genes implicated in regulation of blood vessel development, such as members of the angiopoietin family, were induced in both types of samples leading to significant enrichment of respective ontology terms and pointing to another pathologically relevant process. In line with the results on cell fate changes, this global analysis shows that genes with reduced expression were enriched for terms related to synaptic transmission in both the in vitro model and patient biopsies. In addition, we compared our data to publicly available microarray data from subependymal giant cell astrocytomas (SEGAs) that also arise in TSC patients and show a larger contribution of glial cells as compared to cortical tubers. The correlation in gene expression changes to these glioneural tumors was even stronger (Fig. 2f and Additional file 6: Table S4) with more gene sets passing the significance threshold and showing the same direction of deregulation in both in vitro and in vivo samples. Notably, again inflammation and blood vessel development were the key categories induced, while neuronal differentiation and related terms were those most significantly decreased. Together this analysis indicates that the same type of disease processes is relevant to all three sample types and that our model also molecularly is closer to astrocytomas than cortical tubers.

Of these disease-relevant processes, neuroinflammation in particular is of interest as it is strongly linked to epileptogenesis and likely plays a role in generation of seizures in TSC patients [38]. Thus, we sought to determine levels of inflammatory markers with reported expression in brain lesions by using protein arrays (Additional file 2: Figure S2F). We detected a 12-fold induction of the soluble form of ICAM1, an important indicator for activity of the proinflammatory cascade in astrocytes of TSC lesions [39]. Moreover, there was a nine-fold increase in plasminogen activator uPA that is strongly induced in epileptogenic cortical tubers [40]. Finally, there was a more modest, yet still significant induction of IL6 and IL8 reported for cortical tubers and lung cysts of TSC patients, respectively [41, 42]. Interestingly, inflammatory signaling was recently suggested to be involved in epileptogenesis in a *TSC1* mouse model [43], further highlighting the importance to study inflammatory mechanisms in future efforts to treat TSC-associated epilepsy.

Based on the similarities of physiological processes transcriptionally altered between brain lesion biopsies and our *TSC2* deletion in vitro model, we conclude that our model holds disease relevance and provides valuable insight into the molecular pathology of TSC. In particular, using our comprehensive dataset from the human model system, we could provide deeper insight into the relevant roles of inflammation and angiogenesis in TSC pathology.

### Ribosome profiling demonstrates increased protein synthesis of growth factors in *TSC2*-deficient cells

**Fig. 3:**
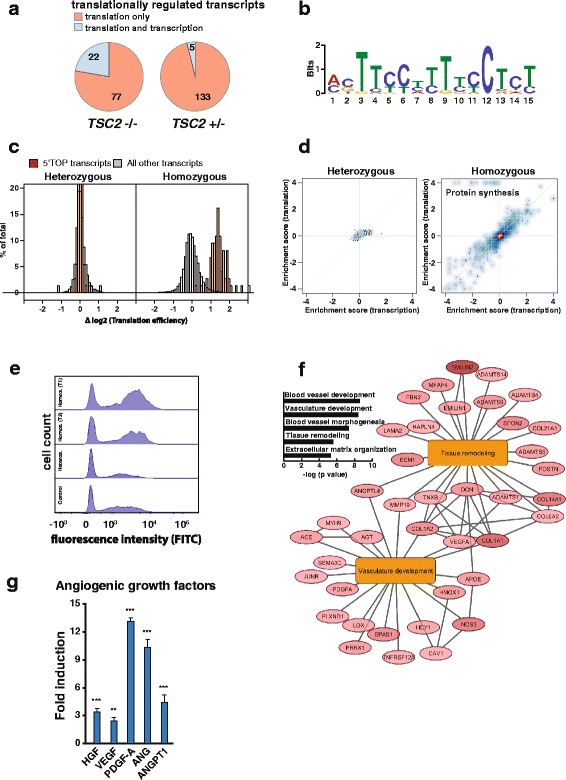
TOP motif mediated enhancement of growth factor synthesis. **a** *Pie chart* representation of the number of transcripts showing a significant change on the level of translation only (*red*; translation changes normalized by transcript level changes) or also on the transcriptional level (*blue*). **b** Detected de novo motif enrichment using the *MEME* tool (*p* < 0.05) within the 5’UTR of genes that showed significant TE changes in *TSC2*-deficient cells. **c** *Density histograms* for log2 changes of TE comparing heterozygous (*left panel*) and homozygous cells (*right*) with control. *Red bars* indicate transcripts with a known 5’-TOP motif and *gray bars* indicate all other detected transcripts. **d** *Scatterplots* of gene set enrichment analysis (GSEA) significance scores comparing transcriptional (*x-axis*) and translational (*y-axis*) changes in *TSC2* heterozygous (*left panel*) and homozygous cells (*right panel*) versus control. Multiple pathways associated with protein synthesis (*labeled*) are positively regulated specifically on the translational level (FDR < 1E-4). **e** Measurement of translational activity in control, *TSC2* heterozygous, and homozygous mutant cells after six weeks of differentiation. Protein synthesis was measured by flow cytometric detection of incorporation of FITC labeled amino acid analog O-propargyl-puromycin into newly synthesized protein. Histograms display the cell count for different levels of OPP incorporation. **f** Network of “vasculature development” and “tissue remodeling” related transcripts that show induction (logFC ≥ 2) of translation in the absence of *TSC2* as determined by Fisher’s exact test (FDR < 0.01). *Ellipse nodes* represent transcripts and *edges* indicate either gene-biological process associations or protein-protein interactions. **g** Detection of angiogenic growth factors in cultures of *TSC2*-deficient and control cell lines (n=3) using protein arrays. Expression levels in control cells are set to 1 and significance of differential expression is determined by Student’s two tailed t-test, *p <0.05, **p <0.01, ***p <0.001

In addition to transcriptional changes, *TSC2*-deficient cells are expected to show translational changes as mTOR signaling is a key regulator of translation initiation [44]. To detect translational dysfunction in our brain lesion model after six weeks of differentiation we performed ribosome profiling, which allows quantification of dynamic changes in translation of all transcripts and can serve as a measure of protein synthesis on a proteome-wide scale by using deep sequencing [45]. In cells homozygous for *TSC2* deletion but also in heterozygous cells, having shown almost no transcriptional changes, translationally deregulated transcripts were detected (Fig. 3a). That the majority of transcripts with changed translation showed no simultaneous change in transcript abundance indicates a regulatory control of translation independent of transcriptional control.

To explore such regulatory control, we performed motif discovery within the 5’UTR of affected transcripts, because mTOR responsiveness of transcripts can be regulated by presence of a terminal oligopyrimidine (TOP) motif in the 5’UTR [46]. Enrichment for such a pyrimidine-rich motif could be confirmed among translationally regulated transcripts in *TSC2* deletion homozygous cells (Fig. 3b), but not in heterozygous cells. The presence of this motif in a transcript was associated with an increase in TE by two-fold to four-fold in *TSC2*-deficient cells, but not for the same transcripts in heterozygous cells (Fig. 3c). To identify overrepresented functional categories associated with mistranslated transcripts and to understand what impact the translational dysfunction may have on the cellular phenotype, we performed gene set enrichment analysis. While no functional category was enriched for the set of transcripts with altered translation in heterozygous cells, a clear and translation level specific enrichment for a function related to protein synthesis was identified in *TSC2* deletion homozygous cells (Fig. 3d, Additional files 7 and 8: Figure S3A and Table S5).

To test if this induction has a direct consequence on the biosynthetic capacity of the cells, we went on to probe protein synthesis rates. Using flow cytometry, the incorporation rate of an amino acid analog into newly synthesized protein was determined and we could confirm that homozygous cells indeed showed higher synthesis rates than heterozygous or control cells (Fig. 3e). To elucidate what biological processes might be affected most we determined their respective enrichment among proteins with a synthesis rate raised by at least four-fold. Proteins with enhanced synthesis rates were most frequently implicated in induction and control of blood vessel formation such as VEGF, PDGF, and AGT (Fig. 3f). This demonstrates that the observed increased transcription of angiogenic factors, recapitulating findings from patient biopsies, leads to higher protein synthesis rates. An additional process highlighted in this analysis was tissue remodeling, which always needs to be closely aligned with regulation of angiogenesis, and their concurrent enrichment points to a co-regulation of these linked processes. To validate these ribosome profiling results we used protein arrays to confirm higher production of angiogenic factors by *TSC2*-deficient cells and we were able to confirm significantly induced levels of the angiogenic growth factors VEGF, HGF, PDGFA, ANG, and ANGPT1 (Fig. 3g and Additional file 9: Table S6).

In summary, we could demonstrate a 5’UTR pyrimidine-rich motif dependent increase in translation of factors involved in protein synthesis in the absence of *TSC2*. Such translation-specific regulation during carcinogenesis was previously also described for transcript subsets implicated in metastasis and regulation of oxidative stress [47, 48]. Here, it illustrates the control of the mTOR pathway over the cell’s protein synthesis capacity, a mechanism frequently exploited by oncogenic events driving hypertrophic growth [49, 50]. Elevated synthesis of angiogenic growth factors in our neural cell-specific in vitro model implies that mutant cells may have the ability to promote blood vessel formation to sustain the growth of *TSC2*-deficient astrocytomas. This notion is also coherent with the highly vascularized tumors of TSC patients in other organs, like angiofibromas and angiomyolipomas [51]. In support of the pro-angiogenic phenotype we observe in our model, it was recently reported that an endothelial-cell-specific deletion of Tsc1 in a mouse model of angiosarcoma led to retinal angiogenesis and formation of vascular tumors [52]. Blocking of autocrine VEGF signaling in this mouse model was able to abolish vascular tumor development and growth. Therefore, inhibition of angiogenesis should also be considered as an alternative treatment strategy for astrocytomas requiring surgery.

### Pharmacological inhibition of mTOR corrects translational defects but not the pathologic cellular state

Having recognized angiogenic growth factors, an inflammatory response, and induced translation of the protein synthesis machinery as altered disease-relevant mechanisms in our TSC model with mTOR hyperfunction, we set out to investigate the therapeutic molecular effects of mTOR inhibitors. To mimic treatment of established brain lesions in TSC patients, we treated *TSC2* deleted cells with mTOR inhibitors after six weeks of differentiation and performed ribosome profiling and RNA-Seq analysis. In clinical trials rapamycin analogs were shown to induce regression of astrocytomas in TSC patients, which however can regrow once treatment is stopped [10, 53]. Besides rapamycin that inhibits mTOR allosterically, we also tested the effect of ATP-competitive mTOR kinase inhibitor AZD-8055 [54].

**Fig. 4:**
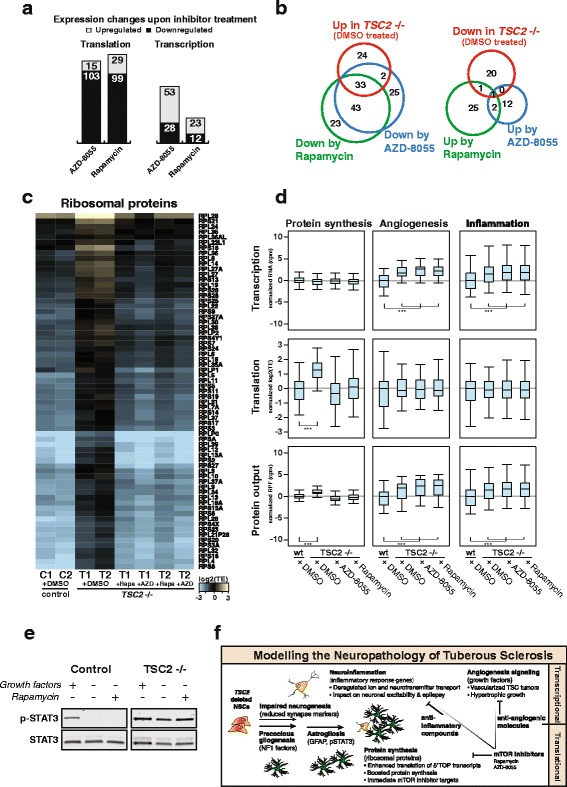
Inhibition of mTOR corrects translational but not transcriptional dysfunction. **a** Number of upregulated (*gray*) or downregulated (*black*) genes at the level of translation or transcription in cells treated with rapamycin or AZD-8055 as compared to vehicle-treated cells. **b** *Venn diagrams* for genes that change in gene expression in opposite directions by *TSC2* loss (*red*) and by compound treatments (*green*, *blue*), respectively. Overlap highlights how many genes deregulated in TSC2 depleted cells (upregulated on *left*, downregulated on *right*) can be reset to control levels by inhibitor treatment. Thresholds of differentially expressed gene calling: abs(logFC) >= 0.5, counts per million no less than 1, and FDR < 0.05. **c** *Heat map* of translational efficiency for transcripts of ribosomal proteins in control cells and *TSC2*-deficient cell line (n = 2) after six weeks of differentiation. Vehicle (DMSO)-treated samples reveal the induced TE in *TSC2* depleted cell lines that is largely reversed after treatment with either rapamycin or AZD-8055. **d** *Box plots* displaying fold changes in transcription, translation, and total protein output for genes associated with proteins synthesis, angiogenesis, and inflammation in control and *TSC2*-deficient cell lines. While different levels of translation of protein synthesis factors between vehicle-treated control and mutant cells are balanced out by mTOR inhibitor treatment, the different transcriptional and protein output for angiogenesis and inflammation genes remains unchanged. Values are log2-transformed and normalized to controls. Statistical significance was determined by a two-tailed paired t-test, ***p < 1×10^−6^. **e** *Western blot analysis* for STAT3 or its phosphorylated form (p-STAT3) from neural cultures after six weeks of differentiation; cultured in the presence (+) or absence (−) of growth factors or rapamycin for the last 12 h before harvest. Elevated phosphorylation even in the presence of rapamycin indicates continued increased pathway activity. **f** Molecular mechanisms of the neuropathology of TSC and new treatment options to be considered

Consistent with the central role of mTOR in activation of translation, treatment with both inhibitors primarily led to reduced translation of transcripts and had a weaker effect on transcription (Fig. 4a and Additional file 10: Table S7). Among the transcripts showing downregulated translation after inhibitor treatment of *TSC2* deleted cells, there were 76 target transcripts shared by both rapamycin and AZD-8055. This shared set represents three-quarters of all transcripts affected in either treatment, suggesting a consistent set of targets affected by mTOR inhibition (Fig. 4b). Importantly, this shared set of translationally downregulated transcripts included more than half of the transcripts that were translationally upregulated in vehicle treated *TSC2* depleted cells as compared to controls, indicating an at least partial rescue of the molecular phenotype.

In accordance with our previous results, these transcripts were essentially all implicated in protein synthesis and mostly coding for ribosomal proteins. When looking specifically at ribosomal transcripts showing induced translation in untreated *TSC2*-deficient cell lines, it was obvious that both rapamycin and AZD-8055 treatment were able to correct elevated translation levels, implying a reversal of the excessive protein synthesis (Fig. 4c and Additional file 11: Table S8). In contrast, increased expression of genes related to angiogenesis and inflammation, both based on induced transcription with no alterations at the level of translation, remained unaffected by treatment with mTOR inhibitors. Accordingly, the respective protein output remained high, even in the drug-treated cells (Fig. 4d). Along these lines, we found that rapamycin treatment also did not reduce STAT3 phosphorylation levels in *TSC2*-deficient cells (Fig. 4e). This suggests that disease mechanisms based on transcriptional changes cannot be treated with mTOR inhibitors even though the initial manifestation of the pathophysiology was dependent on mTOR hyperfunction and future work will address the feasibility and success of combinatorial treatment.

This analysis highlights the importance of monitoring transcription and translation independently when investigating modulation of gene expression by drug treatment. We could demonstrate that both inhibitors have a similar effect and corrected excessive translation of ribosomal proteins with possible implications for reducing hypertrophy of brain tumors in TSC patients. Nonetheless, the transcriptional signature of an inflamed state and angiogenesis remained despite mTOR inhibitor treatment. This shows that while mTOR was effectively inhibited, the cellular pathophysiology was not fully reversed and suggests use of additional combination therapies tailored to the clinical picture of TSC patients (Fig. 4f).

## Discussion

Applying comprehensive genomic analysis to our human stem cell disease model enabled us to gain a deeper understanding of the TSC neuropathology. In TSC2-deficient cultures we detect more cells expressing GFAP, higher levels of STAT3 activation, and increased expression of inflammation associated genes. All these features are indicative of aberrant glial differentiation [34, 36, 55] and are also in agreement with reports on cortical tubers and SEGAs from TSC patients [5, 17, 32, 39]. Importantly, it is now evident that astrocyte pathophysiology can have a detrimental impact on neuronal function as well [56, 57]. Interestingly, an increase in GFAP expressing astrocyte-like cells is equally observed in mouse models for RASopathies including neurofibromatosis, Costello syndrome, Noonan syndrome, and cardio-facio-cutaneous syndrome [58–61]. This illustrates that glial differentiation is frequently perturbed by excessive activation of growth signaling.

Mechanistically of interest for astrocyte dysfunction, appear, in particular, the processes of neuroinflammation and the altered expression of genes regulating glutamate homoestasis. In addition, elevated angiogenic growth factor levels in our neural model suggest enhanced vascularization of brain lesions in analogy to the highly vascularized angiofibromas and angiomyolipomas in TSC patients [51]. As angiogenesis might be a prerequisite for increased synthetic capacity and hypertrophic growth of astrocytomas, it constitutes a possible target for pharmacological intervention in TSC.

Interestingly, in contrast to angiogenic growth factors as in our model of benign hypertrophic astrocytomas, previous work on malignant prostate cancer highlighted metastasis as most significant translationally induced process [47], suggesting that the rerouting of the translational program upon mTOR hyperfunction can be context dependent.

Thus, diseases that could be grouped as “mTORopathies,” due to similar etiology, still present with vastly different phenotypes that require different treatment. As we show in our TSC model, even for a single disease with heterogeneous phenotypes, inhibition of only mTOR signaling may not be sufficient to treat the entire spectrum of pathology. An acquired aberrant cellular state can be independent of mTOR signaling, therefore treatment strategies then need to be tailored to the respective phenotype.

Our stem cell model allows the profiling of molecular alterations present during human embryonic development and then the tracking of aberrant cellular differentiation towards a pathologic state. Further, such a system is well suited to test new treatment strategies as multiple assays can be performed on material derived from the same source in a reproducible fashion using isogenic control lines. These advantages may overcome in many instances the inherent limitations of an in vitro system. As diverse inter-cellular interactions within the brain microenvironment are not exactly recreated, not all disease manifestations may be recapitulated. Conversely, imperfect modeling of physiological conditions can modify the phenotype making comparison to mouse models and patient biopsies, as done here, necessary. Overall, this first human stem cell model of TSC will demonstrate itself to be a valuable system to develop the effective treatment of cellular hyperplasias in TSC patients based on a better understanding of the disease biology.

## Conclusions

Insight into the molecular mechanisms underlying the neuropathology of tuberous sclerosis assists a better mechanistic understanding of the disease and enables development of novel treatment options for the full spectrum of clinical manifestations. By investigating our genome-edited human neural stem cell model, we were able to characterize the molecular pathophysiology by profiling in depth the transcriptional as well as translational changes underlying the disease manifestations. *TSC2*-deficient cells show the transcriptional signature of an inflammatory response with implications for epileptogenesis in TSC patients. In addition, we found intensified translation of ribosomal proteins, which increases biosynthetic capacity in general and boosted production of angiogenic growth factors specifically. Treatment with mTOR inhibitors reset translational dysfunction but was incapable to correct transcription-based molecular pathologies. Importantly, in addition to the detailed description of the molecular pathology, we highlight novel entry points for tailored pharmacological therapies that hold potential to provide substantial benefits to patients suffering from TSC in the future.

## Additional files

Additional file 1: Figure S1. Deregulated expression of neuronal and glial markers in the absence of *TSC2*. (PDF 2.73 mb) Additional file 2: Figure S2. Loss of *TSC2* triggers expression changes related to inflammatory response, metabolism, and neuronal function. (PDF 1.15 mb) Additional file 3: Table S1. RNA-Seq and ribosome profiling data of differentiated *TSC2* wild-type, heterozygous, and homozygous deletion cell lines (related to Figs. 2 and 3). (XLSX 6.88 mb) Additional file 4: Table S2. Gene set enrichment analysis of RNA-Seq data (related to Additional file 2: Figure S2B and E). (XLSX 22.2 kb) Additional file 5: Table S3. Gene ontology term enrichments for RNA-Seq data from differentiated *TSC2* deletion cell lines and microarray data of patient cortical tubers (related to Fig. 2e). (XLSX 19.8 kb) Additional file 6: Table S4. Gene ontology term enrichments for RNA-Seq data from differentiated *TSC2* deletion cell lines and microarray data of patient SEGAs (related to Fig. 2f). (XLSX 27.7 kb) Additional file 7: Figure S3. Loss of *TSC2* increases the TE of factors implicated in protein synthesis. (PDF 417 kb) Additional file 8: Table S5. Gene set enrichment analysis of ribosome profiling data (related to Fig. 3d). (XLSX 11.1 kb) Additional file 9: Table S6. Gene ontology term enrichments for ribosome profiling data (related to Fig. 3f). (XLSX 11 kb) Additional file 10: Table S7. RNA-Seq and ribosome profiling data of differentiated *TSC2* wild-type and homozygous deletion cell lines treated with mTOR inhibitors (related to Fig. 4). (XLSB 7.52 mb) Additional file 11: Table S8. Gene sets and associated genes that show after mTOR treatment a reversal of the change in expression detected in untreated *TSC2* deletion cells (related to Fig. 4b). (XLSX 25.8 kb)

## Abbreviations

ATP: Adenosine triphosphate
BCA: Bicinchoninic assay
cDNA: Complementary DNA
DAPI: 4’,6-diamidino-2-phenylindole
DMSO: Dimethyl sulfoxide
DNA: Desoxyribonuceic acid
ECL: Enhanced chemiluminescence
ESC: Embryonic stem cell
FDR: False discovery rate
GSEA: Gene set enrichment analysis
HRP: Horseradish peroxidase
LOH: Loss of heterozygosity
mTOR: Mechanistic target of rapamycin
NSC: Neural stem cell
OPP: O-propargyl-puromycin
PBS: Phosphate buffered saline
PCR: Polymerase chain reaction
PFA: Paraformaldehyde
qPCR: Quantitative PCR
RNA: Ribonucleic acid
RNA-Seq: RNA-Sequencing
rRNA: Ribosomal RNA
SDS-PAGE: Sodium dodecyl sulphate-polyacrylamide gel electrophoresis
SEGA: Subependymal giant cell astrocytoma
TOP: Terminal oligo-pyrimidine
tRNA: Transfer RNA
TSC: Tuberous sclerosis complex
UTR: Untranslated region
