# Supplementary material for: Genomic analysis of the molecular neuropathology of tuberous sclerosis using a human stem cell model
Source: Genome Med. 2016 Sep 21;8:94. doi: 10.1186/s13073-016-0347-3 (PMC5031259; doi:10.1186/s13073-016-0347-3)
Supplement: Additional file 1: Figure S1. — Deregulated expression of neuronal and glial markers in the absence of TSC2. (PDF 2.73 mb) [file 13073_2016_347_MOESM1_ESM.pdf]

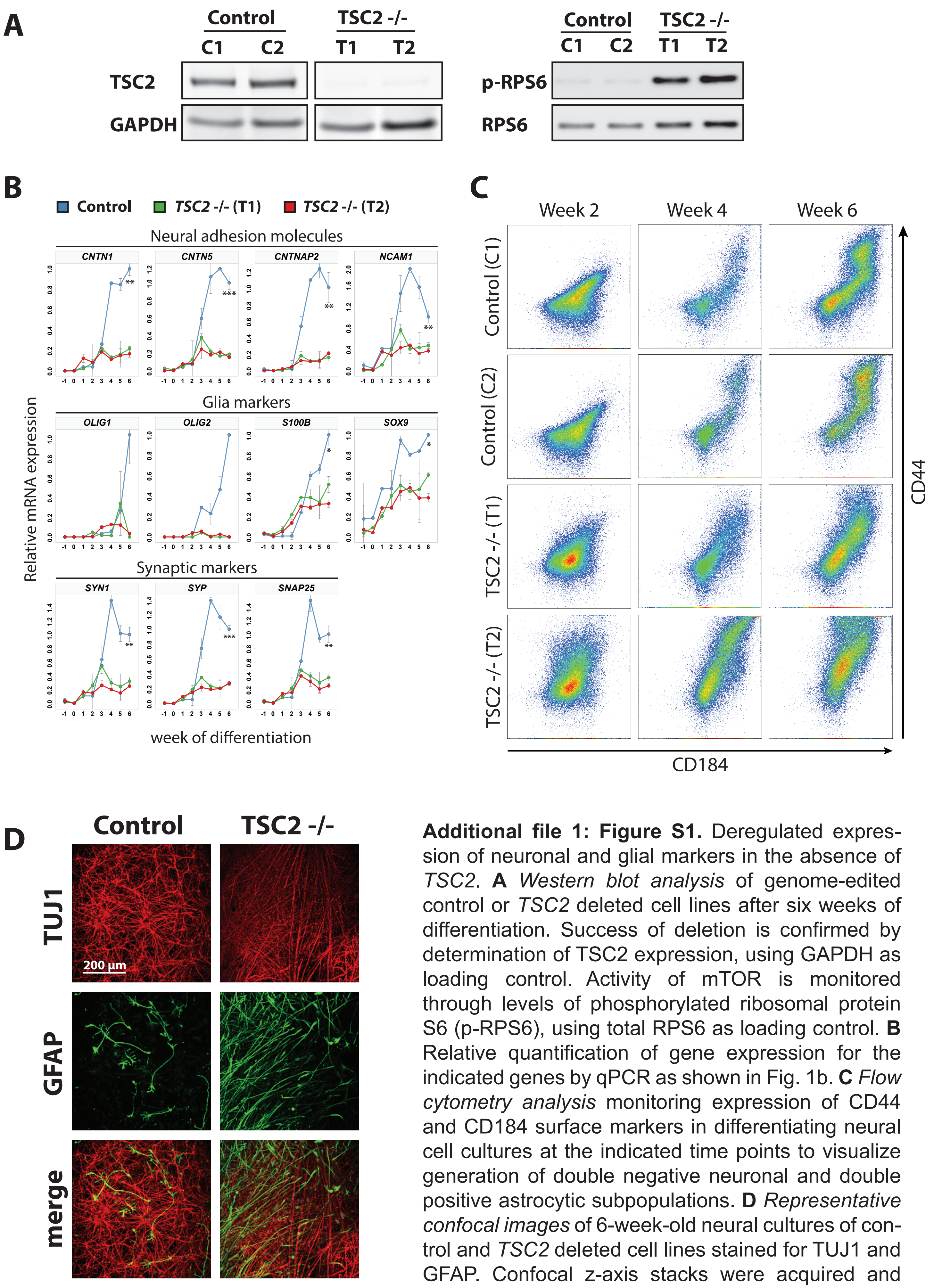

Additional file 1: Figure S1. Deregulated expression of neuronal and glial markers in the absence of *TSC2*. **A** *Western blot analysis* of genome-edited control or *TSC2* deleted cell lines after six weeks of differentiation. Success of deletion is confirmed by determination of TSC2 expression, using GAPDH as loading control. Activity of mTOR is monitored through levels of phosphorylated ribosomal protein S6 (p-RPS6), using total RPS6 as loading control. **B** Relative quantification of gene expression for the indicated genes by qPCR as shown in Fig. 1b. **C** *Flow cytometry analysis* monitoring expression of CD44 and CD184 surface markers in differentiating neural cell cultures at the indicated time points to visualize generation of double negative neuronal and double positive astrocytic subpopulations. **D** *Representative confocal images* of 6-week-old neural cultures of control and *TSC2* deleted cell lines stained for TUJ1 and GFAP. Confocal z-axis stacks were acquired and restructured.
