# Supplementary material for: Genomic analysis of the molecular neuropathology of tuberous sclerosis using a human stem cell model
Source: Genome Med. 2016 Sep 21;8:94. doi: 10.1186/s13073-016-0347-3 (PMC5031259; doi:10.1186/s13073-016-0347-3)
Supplement: Additional file 2: Figure S2. — Loss of TSC2 triggers expression changes related to inflammatory response, metabolism, and neuronal function. (PDF 1.15 mb) [file 13073_2016_347_MOESM2_ESM.pdf]

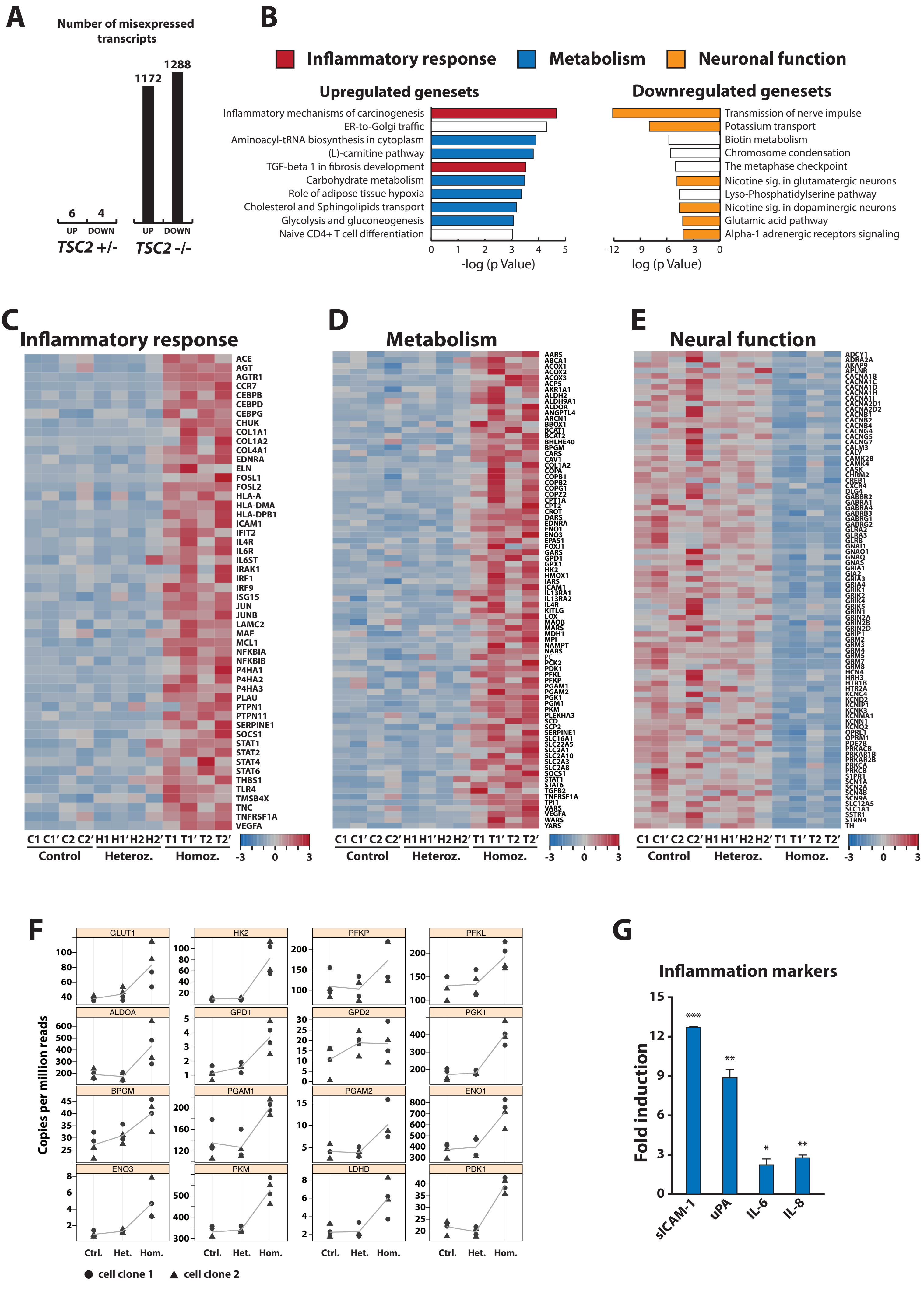
A
Number of misexpressed transcripts
6
4
UP
DOWN
TSC2 +/-
1172
1288
UP
DOWN
TSC2 -/-
B
Inflammatory response
Metabolism
Neuronal function
Upregulated genesets
Inflammatory mechanisms of carcinogenesis
ER-to-Golgi traffic
Aminoacyl-tRNA biosynthesis in cytoplasm
(L)-carnitine pathway
TGF-beta 1 in fibrosis development
Carbohydrate metabolism
Role of adipose tissue hypoxia
Cholesterol and Sphingolipids transport
Glycolysis and gluconeogenesis
Naive CD4+ T cell differentiation
-log (p Value)
Downregulated genesets
Transmission of nerve impulse
Potassium transport
Biotin metabolism
Chromosome condensation
The metaphase checkpoint
Nicotine sig. in glutamatergic neurons
Lyso-Phosphatidylserine pathway
Nicotine sig. in dopaminergic neurons
Glutamic acid pathway
Alpha-1 adrenergic receptors signaling
log (p Value)
C
Inflammatory response
D
Metabolism
E
Neural function
C1 C1′ C2 C2′ H1 H1′ H2 H2′ T1 T1′ T2 T2′
Control
Heteroz.
Homoz.
F
GLUT1
HK2
PFKP
PFKL
ALDOA
GPD1
GPD2
PGK1
BPGM
PGAM1
PGAM2
ENO1
ENO3
PKM
LDHD
PDK1
Copies per million reads
Ctrl.
Het.
Hom.
cell clone 1
cell clone 2
G
Inflammation markers
Fold induction
sICAM-1
uPA
IL-6
IL-8

Additional file 2: Figure S2. Loss of *TSC2* triggers expression changes related to inflammatory response, metabolism, and neuronal function. **A** *Bar graphs* representing the total number of transcripts significantly upregulated or downregulated in *TSC2* heterozygous and homozygous mutant cells as compared to wild-type lines. Threshold: Benjamini–Hochberg adjusted p value < 0.05. **B** *Bar charts* showing biological processes significantly enriched for genes upregulated and downregulated in *TSC2* depleted cells as determined by gene set enrichment analysis. **C** *Heat map* of expression levels of genes associated with “inflammatory response” in the indicated cell lines and biological replicates. Gene expression levels are normalized to have zero average and unit (one) standard variance. **D** Expression levels of genes associated with “metabolism,” as shown in (**C**). **E** Expression levels of genes associated with “neural function,” as shown in (**C**). **F** *Dot plots* for examples of genes involved in the glycolytic pathway (displayed in Fig. 3g). Data are shown for biological replicates of two independent cell clones (*circle*, *triangle*) of the indicated genotypes. *Solid lines* indicate average values of all replicates. **G** Expression of proteins indicative of inflammation in control and *TSC2*-deficient 6-week-old cell cultures (n = 3) determined by a human protein array. Expression levels in control cells are set to 1 and significance of differential expression is determined by Student’s two tailed t-test, $^*p < 0.05$, $^{**}p < 0.01$, $^{***}p < 0.001$.
