# Supplementary material for: Genomic analysis of the molecular neuropathology of tuberous sclerosis using a human stem cell model
Source: Genome Med. 2016 Sep 21;8:94. doi: 10.1186/s13073-016-0347-3 (PMC5031259; doi:10.1186/s13073-016-0347-3)
Supplement: Additional file 7: Figure S3. — Loss of TSC2 increases the TE of factors implicated in protein synthesis. (PDF 417 kb) [file 13073_2016_347_MOESM7_ESM.pdf]

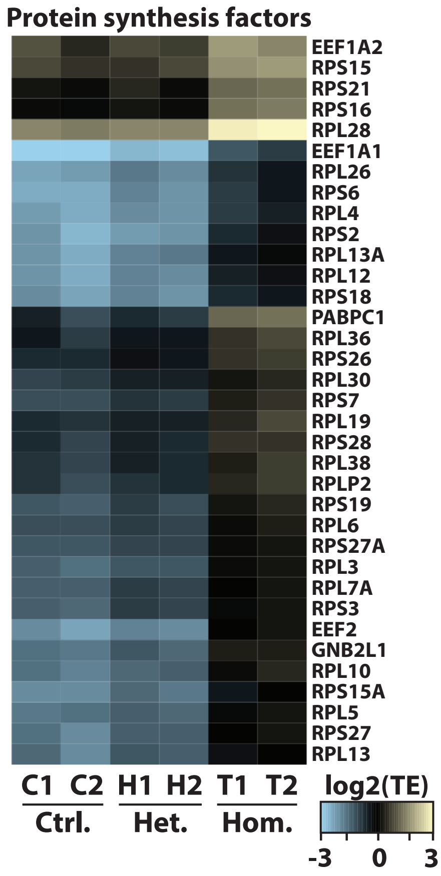

Additional file 7: Figure S3. Loss of *TSC2* increases the TE of factors implicated in protein synthesis. *Heat map* of TE of coding for protein synthesis factors in control, *TSC2* heterozygous and homozygous mutant cell lines (n = 2) after 6 weeks of differentiation.
